# Recent Insights into the Mitochondrial Role in Autophagy and Its Regulation by Oxidative Stress

**DOI:** 10.1155/2019/3809308

**Published:** 2019-11-04

**Authors:** Vicente Roca-Agujetas, Cristina de Dios, Laura Lestón, Montserrat Marí, Albert Morales, Anna Colell

**Affiliations:** ^1^Department of Cell Death and Proliferation, Institut d'Investigacions Biomèdiques de Barcelona, Consejo Superior de Investigaciones Científicas (IIBB-CSIC), Institut d'Investigacions Biomèdiques August Pi i Sunyer (IDIBAPS), Barcelona, Spain; ^2^Centro de Investigación Biomédica en Red sobre Enfermedades Neurodegenerativas (CIBERNED), Spain; ^3^Departament de Biomedicina, Facultat de Medicina, Universitat de Barcelona, Barcelona, Spain

## Abstract

Autophagy is a self-digestive process that degrades intracellular components, including damaged organelles, to maintain energy homeostasis and to cope with cellular stress. Autophagy plays a key role during development and adult tissue homeostasis, and growing evidence indicates that this catalytic process also has a direct role in modulating aging. Although autophagy is essentially protective, depending on the cellular context and stimuli, autophagy outcome can lead to either abnormal cell growth or cell death. The autophagic process requires a tight regulation, with cellular events following distinct stages and governed by a wide molecular machinery. Reactive oxygen species (ROS) have been involved in autophagy regulation through multiple signaling pathways, and mitochondria, the main source of endogenous ROS, have emerged as essential signal transducers that mediate autophagy. In the present review, we aim to summarize the regulatory function of mitochondria in the autophagic process, particularly regarding the mitochondrial role as the coordination node in the autophagy signaling pathway, involving mitochondrial oxidative stress, and their participation as membrane donors in the initial steps of autophagosome assembly.

## 1. Introduction

Autophagy (literally “self-eating” in Greek) is a cellular catabolic process that delivers cytoplasm constituents including macromolecules and damaged organelles to lysosomes for degradation [1, 2]. Three types of autophagy have been described, namely, macroautophagy, microautophagy, and chaperone-mediated autophagy that differ in their way of cargo transportation and regulation. Macroautophagy (here after referred to as autophagy) is the best characterized and, unlike microautophagy and chaperone-mediated autophagy, involves the formation of a double membrane vesicle called autophagosome.

Autophagy occurs at basal levels to preserve cellular homeostasis by recycling proteins and organelles [1, 2]. This catabolic process can also act in response to cellular insults such as nutrient or growth factor deprivation, hypoxia, and oxidative stress. During periods of starvation, autophagy degrades cytoplasmic materials to produce amino acids and fatty acids that can be used to synthesize new proteins or to produce ATP for cell survival. However, when autophagy is excessively induced, it can result in cell death [3]. The autophagic pathway has to be tightly regulated. Too little or too much autophagy can be deleterious, and in fact, dysregulation of autophagy is underlying in a wide range of diseases including neurodegenerative disorders most typically involving the accumulation of pathogenic proteins, inflammatory disorders such as Crohn disease, and cancer [4, 5]. The regulation of autophagy has been extensively studied in the past few years, and several reviews have thoroughly summarized the progress in this area [6–9]. In this review, we will focus on the recent studies that highlight the role of mitochondria and mitochondrial oxidative stress in autophagy regulation. For a better understanding of the mechanisms involved in this process, we will first outline the key elements that govern the autophagy pathway.

## 2. Cell Signaling Pathways of Autophagy

**Figure 1:**
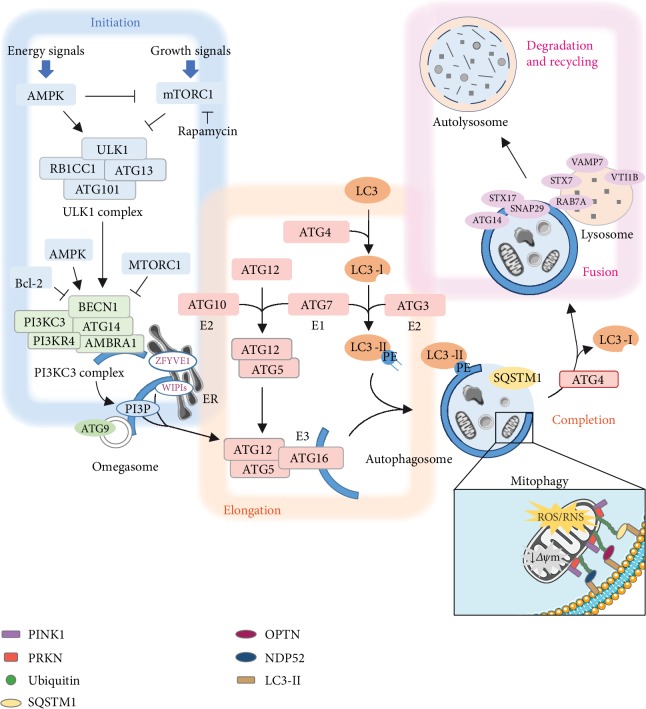
Schematic process of the canonical autophagy pathway. Upon deprivation of nutrients or growth signals, the activation of AMPK and/or the inhibition of mTORC1 fosters the initiation of autophagy via regulation of ULK1 and PI3KC3 complexes. ULK1 needs ATG13, RB1CC1, and ATG101 to phosphorylate BECN1, which disrupts the inhibitory association with the antiapoptotic protein Bcl-2 and allows for PI3KC3 complex assembly. The PI3KC3 complex, in the newly formed membranes, known as omegasomes, phosphorylates phosphatidylinositol to form PI3P. During the elongation phase, MAP1LC3/LC3 incorporation to the preautophagosomal membranes requires the involvement of different ubiquitin-related enzymes: the ubiquitin-activating and conjugating enzymes ATG7 (E1), and ATG10 (E2) catalyze the association between the ubiquitin-like protein ATG12 and ATG5; at the same time, the protease ATG4 cleaves pro-MAP1LC3/LC3, allowing ATG7 (E1 ubiquitin-activating enzyme) and ATG3 (E2 ubiquitin-conjugating enzyme) catalyze MAP1LC3/LC3-I conjugation with PE. Next, ATG16 (E3 ubiquitin-protein ligase) stabilizes the ATG12-ATG5 complex and facilitates the lipidated MAP1LC3/LC3-II localization to membranes. Completion stage involves protein SQSTM1/p62, which acts as a bridge between polyubiquitinated cargo and MAP1LC3/LC3-II-autophagosomes. Once autophagosome maturation is finished, ATG4 catalyzes the deconjugation of MAP1LC3/LC3-II to MAP1LC3/LC3-I. Different proteins participate in the fusion events between autophagosomes and lysosomes including the target-membrane-bound (t) SNARE proteins STX17 and SNAP29, the vesicle-localized (v) SNARE VTI1B, the beclin1-associated regulator ATG14, and the lysosomal membrane proteins RAB7B, VAMP7, and STX7. Finally, the fusion results in the autolysosome with the subsequent degradation and recycling of the cellular components. Magnified section in autophagosome depicts the PINK1-PRKN pathway of mitophagy. Following a mitochondrial stress, PINK1 accumulates in the OMM of depolarized mitochondria. Autophosphorylation stabilizes PINK1 and elicits the translocation of the E3 ubiquitin ligase PRKN from cytosol. Activated PRKN elongates and conjugates ubiquitin chains on different OMM proteins. K63-linked ubiquitin chains serve as a signal for the recruitment of mitophagy receptors such as SQSTM1, optineurin (OPTN), and NDP52, which interact with LC3-II and mediate autophagosome initiation on the damaged mitochondrion. *ΔΨ*m: mitochondrial membrane potential.

Autophagy follows a sequential course that starts with the formation of an isolation membrane, the phagophore, that elongates and seals on itself [4, 10]. The nascent double membrane vacuole, known as autophagosome, fuses with a lysosome and the enclosed cargo is degraded by the lysosomal proteases. The entire pathway is coordinated by autophagy-related protein (ATG) in a highly regulated manner. A major player in this process is the unc-51-like kinase 1 (ULK1) complex composed by ULK1, ATG13, ATG101, and the RB1-inducible coiled-coil protein 1 (RB1CC1/FIP200). The ULK1 complex is regulated by the nutrient-sensing mechanistic target of rapamycin complex 1 (mTORC1) and by the AMP-activated protein kinase (AMPK), whose activation depends on the cellular energy status [9, 11]. The complex integrates the upstream nutrient and energy signals to coordinate the initiation of autophagy (Figure 1). Under nutrient-replete conditions, mTORC1 phosphorylates ULK1 and ATG13, which prevents the activation of the ULK1 complex, likely through a specific conformational change [8, 12]. Instead, an intracellular increase of AMP, concomitant to decreased availability of ATP, activates AMPK, which in turn catalyzes the activating phosphorylation of ULK1. AMPK can also act inhibiting the mTORC1 signaling pathway through direct phosphorylation of the regulatory-associated protein of mTOR (RPTOR/raptor) or via the activating phosphorylation of the mTORC1 inhibitor tuberous sclerosis complex subunit 2 (TSC2) [13, 14].

Nucleation of the preautophagosomal structures requires the participation of the phosphatidylinositol 3-kinase catalytic subunit type 3 (PI3KC3/VPS34) complex (Figure 1), integrated by the catalytic subunit, PI3KC3/VPS34 kinase, the scaffold protein phosphoinositide-3-kinase regulatory subunit 4 (PI3KR4/VPS15) kinase, and beclin 1 (BECN1) [11]. AMPK phosphorylates BECN1 and PI3KC3/VPS34 subunits stimulating the autophagic functions of the PI3KC3 complexes [15]. Activated ULK1 can also phosphorylate BECN1 on Ser 14, thereby enhancing the lipid kinase activity of the PI3KC3 complex and producing phosphatidylinositol-3-phosphate (PI3P) [16]. The local PI3P increase at the endoplasmic reticulum (ER) membranes is concomitant with an ATG9-dependent membrane acquisition from endosomal vesicles [17] and triggers the recruitment of PI3P-binding effectors, including zinc finger FYVE-type containing protein 1 (ZFYVE1/DFCP1) and the WD repeat domain, phosphoinositide interacting (WIPI) proteins [18, 19]. ZFYVE1/DFCP1 binds to PI3P giving rise to an ER structure called the omegasome that serves as a scaffold for the phagophore formation [19]. The elongation step of the phagophore in turn depends on the interaction of PI3P with WIPI2 [20].

Different molecules have been reported to bind BECN1 and determine its specific role in autophagy [21]. The binding of BECN1 to ATG14 and the activating molecule in BECN1-regulated autophagy protein 1 (AMBRA1) targets the PI3KC3 complex to the sites of phagophore biogenesis, whereas the recruitment of endophilin B1, which associates indirectly with BECN1 via UV radiation resistance-associated protein (UVRAG) seems to be involved in the endocytic trafficking and the autophagosomal maturation. The positive regulatory effect of UVRAG is counteracted by the Run domain Beclin-1-interacting and cysteine-rich domain-containing protein (RUBCN/rubicon). Different reports show that mTORC1 inhibits the PI3KC3 complex through phosphorylation of the regulatory subunits ATG14, AMBRA1, or UVRAG [22–24]. It is also well established that the antiapoptotic Bcl-2 family members can suppress PI3KC3 complex activity by direct binding to BECN1. Phosphorylation of Bcl-2 and BECN1 disrupts their interaction and releases BECN1 for autophagy [25].

**Figure 2:**
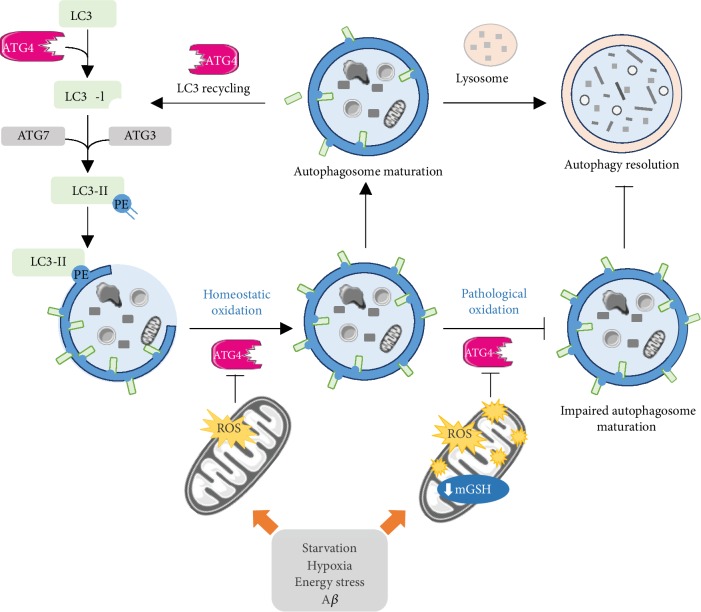
Redox regulation of ATG4 activity. The cysteine protease ATG4 participates in autophagosome formation at the elongation and completion steps. This enzyme facilitates the conjugation of MAP1LC3/LC3, through pro-MAP1LC3/LC3 cleavage in MAP1LC3/LC3-I, and the posterior deconjugation and recycling of MAP1LC3/LC3. This process is carefully regulated by the ATG4 redox state. A transient oxidation of ATG4 inhibits its proteolytic activity, thereby facilitating autophagosome maturation and the subsequent autophagy resolution. In contrast, under pathological situations, such as excessive starvation, hypoxia, high energy stress, or A*β* toxicity, the increased mitochondrial oxidative stress results in higher ATG4 oxidation that prevents a proper LC3 recycling and impairs autophagosome maturation. Depletion of the mitochondrial GSH content, the main mitochondrial antioxidant defense, stimulates the mitochondrial ROS generation resulting in an enhanced inhibitory effect of ATG4 proteolytic activity.

The recruitment of the cargo and the expansion and closure of the phagophore membranes are mediated by two ubiquitin-like molecules, ATG12 and the Atg8 family proteins (mammalian homologs of yeast Atg8), which in turn are split into two subfamilies: the microtubule-associated proteins 1A/1B light chain 3 (MAP1LC3/LC3) family and the GABA type A receptor-associated protein (GABARAP) family [10, 11] (Figure 1). In both cases, the protein undergoes an ubiquitin-like conjugation reaction that requires first its activation by the E1-like enzyme ATG7. Then, ATG12 is covalently linked to ATG5 by ATG10, an E2-like ubiquitin carrier protein, while the Atg8 family proteins are transferred to the E2-like enzyme ATG3 and conjugated to the phosphatidylethanolamine (PE) present in the phagosome membranes. This final conjugation step is catalyzed by the ATG12eATG5-ATG16L1 complex and requires the previous processing of the Atg8 family protein (MAP1LC3/LC3 or GABARAP) by ATG4, which cleaves its C-terminal region, exposing a key glycine residue that forms a covalent bond with the amine of the PE headgroup (Figure 2). The lipidated form of MAP1LC3/LC3, referred to as LC3-II, has been reported to recruit proteins containing an LC3-interacting region (LIR). Some of these LIR-containing proteins facilitate the phagophore expansion and closure, while others act as receptors, conveying cargo specificity to the growing phagophore. ATG4 can also delipidate the conjugated protein, releasing MAP1LC3/LC3 or other Atg8 family proteins from the autophagosome membranes (Figure 2) and, at least in yeast, allowing the elongation of the autophagosome [26].

Autophagosomes can either engulf intracellular material in a nonselective manner or deliver specific organelles and proteins, depending on the initiating stimulus [27]. Several receptors participate in the selective recognition and recruitment of autophagosomal cargo. The best characterized autophagy adaptor is the sequestosome 1 (SQSTM1/p62), which interacts noncovalently with ubiquitin or polyubiquitin chains via the ubiquitin-associated domain and delivers the polyubiquitinated cargoes to autophagy via its LIR region [28]. The lysosome-mediated turnover of SQSTM1/p62 has widely been used to monitor the autophagic flux.

The selective engulfment of damaged or superfluous mitochondria into autophagosomes, known as mitophagy, is mainly driven by the parkin RBR E3 ubiquitin ligase (PRKN) and the PTEN-induced kinase 1 (PINK1) [29] (Figure 1), although different pathways of PRKN-independent mitophagy have also been described [30]. In particular, following a stress, damaged mitochondria lose their transmembrane potential (*ΔΨ*) and PINK1 accumulates in the outer membrane of mitochondria (OMM) where it phosphorylates ubiquitin at Ser65 to activate PRKN activity (Figure 1). Once activated, PRKN elongates and conjugates ubiquitin chains on OMM proteins, which in turn recruit mitophagy receptors such as SQSTM1/p62, optineurin (OPTN), and calcium binding and coiled-coil domain 2 (CALCOCO2/NDP52), all of them containing a LIR motif to interact with the MAP1LC3/LC3 anchored in the autophagosome membrane.

Once autophagosomes are formed, they can fuse with lysosomes or late endosomes to form amphisomes, which ultimately fuses with lysosomes [31] (Figure 1). Notably, autophagosomes are not able to fuse with lysosomes until the ATG machinery at their surface has disassembled [32]. Furthermore, vesicles have to move closer together first to become tethered afterwards [33]. Besides cytoskeleton components and related motor proteins, the membrane fusion process requires a conserved machinery that consists of RAB GTPases, membrane-tethering effectors that mediate the first contact, and specific soluble NSF attachment protein receptors (SNAREs), such as syntaxin 7 (STX7), syntaxin 17 (STX17), synaptosome-associated protein 29 (SNAP29), vesicle-associated membrane protein 7 (VAMP7), and vesicle transport through interaction with t-SNAREs 1B (VTI1B) protein, which have been directly or indirectly implicated in the autophagosome- and endosome-lysosome fusion process [34]. Recent studies have also identified ATG14 as a key player in autophagosome and lysosome fusion, through its binding and subsequent stabilization of the STX17-SNAP29 complex on the autophagosome membranes [35]. The fusion process concludes after the elimination of the inner autophagosomal membrane by lysosomal hydrolases. Degradation of the autophagosome content proceeds as the lysosomal lumen is acidified owing to the activity of the V-type ATPase [36].

## 3. Regulation of Autophagy by Mitochondrial Oxidative Stress

**Figure 3:**
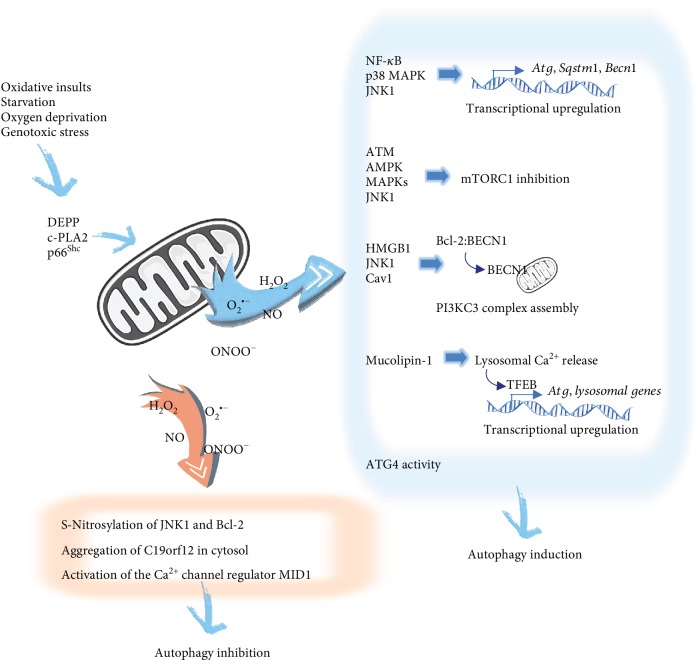
Cross talk between induction of mitochondrial oxidative/nitrosative stress and autophagy. Oxidative insults, starvation, oxygen deprivation, and genotoxic stress inducers, among others, activate different upstream signaling pathways and signaling effectors such as DEPP, KAT5/c-PLA2, and the redox-sensitive kinase p66^Shc^ that converge on mitochondria and trigger ROS and RNS production. Downstream signaling of mitochondrial stress-mediated autophagy induction occurs at different steps of the autophagy pathway. Transcriptional upregulation of *Sqstm1*, *Becn1*, and some *Atg* genes has been reported via oxidative stress-mediated phosphorylation of NF-*κ*B and the induction of p38 MAPK and JNK. Autophagy induction has also been described by mTORC1 inhibition after ROS/RNS-mediated activation of ATM-AMPK, MAPK, and JNK1 pathways. Under ROS/RNS accumulation, HMGB1 and JNK1 promote the disruption of the inhibitory effect of Bcl-2 in BECN1, facilitating BECN1 translocation to mitochondria. BECN1 translocation and PI3KC3 complex assembly are also triggered by H_2_O_2_-mediated phosphorylation of caveolin 1 (Cav1). Mitochondrial ROS-mediated activation of the lysosomal Ca^2+^ channel mucolipin-1 triggers a Ca^2+^-mediated signaling pathway that upregulates some *Atg* and lysosomal genes. Mitochondrial H_2_O_2_ promotes autophagosome synthesis by a transient oxidative inhibition of ATG4 activity (as described in detail in Figure 2). Meanwhile, under certain conditions, mitochondrial stress has likewise been reported as an autophagy suppressor. ROS and RSN, as autophagy inhibitor mediators, have been associated to the S-nitrosylation of JNK1 and Bcl-2, the aggregation of the mitochondrial membrane protein C19orf12 in cytosol, and the activation of the Ca^2+^ channel regulatory protein MID1.

A growing body of work suggests that reactive oxygen species (ROS) are important cellular signal transducers controlling autophagy during nutrient starvation [37]. It is, however, still a matter of debate which species are involved. While Chen et al. [38] have reported superoxide (O_2_^•-^) as the primary ROS involved in autophagy induced by nutritional deprivation, other works indicate that hydrogen peroxide (H_2_O_2_) is the molecule produced immediately after starvation [39]. A similar link has been described between reactive nitrogen species (RNS) and autophagy under ischemic injury [40], showing that peroxynitrite (ONOO^−^), induced by oxygen-glucose deprivation, can trigger autophagy. Moreover, it has been reported that, once deprived of nutrients, cells actively extrude GSH in order to shift an intracellular redox environment toward more oxidizing conditions and prime redox-sensitive proteins involved in both induction and execution of autophagy [41]. Remarkably, these studies show that the sole chemically induced oxidation of GSH is able to trigger autophagy, even in the absence of any autophagic stimulus, hence, underlying the importance of thiol redox homeostasis in autophagy commitment. Similarly, stimuli like tumor necrosis factor alpha (TNF-*α*) and lipopolysaccharides (LPS) engage ROS/RNS generation signaling pathways that, in turn, can induce autophagy [42, 43]. In most of these studies, mitochondria are the major source of ROS/RNS for autophagy induction. It has been reported that under genotoxic stress the decidual protein induced by progesterone (DEPP/C10orf10), a transcriptional target of forkhead box O3 (FOXO3), localizes in mitochondria, promoting mitochondrial ROS/RNS accumulation and formation of autophagosomes [44] (Figure 3). Autophagy is blocked after incubation with N-acetyl cysteine and the superoxide dismutase mimetic and ONOO^−^ scavenger manganese (III) tetrakis (4-benzoic acid)porphyrin (MnTBAP), which localizes to the mitochondria and, therefore, further supports the notion that mitochondrial oxidative/nitrosative stress contributes to DEPP-triggered autophagy. [44]. Interferon-*γ* has also been shown to promote autophagy-associated apoptosis via inducing lysine acetyltransferase 5- (KAT5/cPLA2-) dependent mitochondrial ROS production assessed by using the MitoSOX Red mitochondrial superoxide indicator [45] (Figure 3). Additionally, under stress conditions like ethanol exposure, cells respond by activating the mitochondrial fission machinery in a manner that stimulates protective autophagy through mitochondrial ROS [46]. The ethanol-induced autophagic response decreases after blocking free radical production using the mitochondria-directed antioxidant agent MitoQ (mitoquinone), a compound known to scavenge lipid peroxyl radicals, ONOO^−^, and O_2_^•-^ [47]. In turn, autophagy inhibition increases mitochondrial fission and cell death [46]. Recently, the redox-sensitive kinase p66^Shc^, a member of the Shc family of adaptor proteins and critical regulator of longevity [48], has been identified as a mediator of autophagy [49, 50]. Upon phosphorylation, p66^Shc^ translocates to the intermembrane space of the mitochondria where it oxidizes cytochrome c and catalyzes the partial reduction of O_2_ to H_2_O_2_ [48] (Figure 3). The binding between p66^Shc^ and cytochrome c triggers AMPK-mediated autophagy and mitophagy, through impairing the mitochondrial function and lowering the mitochondrial ATP production [50]. Conversely, silencing of p66^Shc^ has been shown to prevent nutrient starvation-induced autophagy and increase apoptosis resistance [49].

The autophagic pathway is regulated by ROS/RNS at different levels and through multiple mechanisms. In retinal pigmented epithelial cell, H_2_O_2_-mediated NF-*κ*B phosphorylation has been shown to stimulate SQSTM1/p62 and ATG10 expression [51] (Figure 3). ATG genes can also be upregulated by ROS-induced p38 mitogen-activated protein kinase (p38 MAPK) and c-Jun N-terminal kinase 1 (JNK1) [52, 53] (Figure 3). Further work in *Drosophila* has shown that JNK-mediated autophagy is engaged after paraquat-induced mitochondrial oxidative stress and requires the interaction between ATG9 and tumor necrosis factor receptor-associated factor 2 (dTRAF2) [54]. Notably, some chemotherapeutic agents have been described to trigger autophagic cell death in tumor cells through ROS-dependent suppression of the mTORC1 signaling pathway [55, 56], involving mitochondria and different MAPK pathways [57, 58] (Figure 3). mTORC1 activity can be directly inhibited by oxidative stress [59] or through nitric oxide- (NO-) and H_2_O_2_-induced activation of the ATM-AMPK pathway [60–62] (Figure 3). Oxidative stress can also stimulate PI3KC3 complex assembly. In cancer cells, mitochondrial ROS produced after low-power laser irradiation have been shown to upregulate BECN1 expression via the rise of p65/RELA transcriptional activity [63] (Figure 3). Furthermore, it has been described that the inhibition of the mitochondrial electron transport chain—with the consequent generation of ROS—promotes the translocation of high mobility group box 1 (HMGB1) protein from the nucleus to the cytosol, where it disrupts the inhibitory interaction of Bcl-2 with BECN1 [64] (Figure 3). Release of BECN1 and autophagy induction are also observed after phosphorylation of Bcl-2 by JNK1 [65] (Figure 3), which becomes activated following the oxidation of its upstream redox-sensitive regulator mitogen-activated protein kinase kinase kinase 5 (MAP3K5/ASK1) [66]. Recent studies demonstrate that in response to H_2_O_2_, phosphorylated caveolin 1 promotes the translocation of BECN1 to mitochondria and facilitates autophagosome formation by interacting with the PI3KC3 complex through its scaffolding domain [67] (Figure 3). Of note, there is also opposite evidence indicating that ROS/RNS suppress autophagy instead of promoting its activation. For instance, Venco et al. [68] have shown that oxidative stress inhibits the autophagy observed after overexpression of the mitochondrial membrane protein C19orf12, most likely by favoring its aggregation in cytosol (Figure 3). NO-dependent inhibition of autophagy has also been reported, by S-nitrosylation of Bcl-2 or via S-nitrosylation and inactivation of JNK1, which leads to a reduction of Bcl-2 phosphorylation and, in turn, increases Bcl-2-BECN1 interaction [69, 70] (Figure 3).

It is increasingly evident that the relationship between ROS and Ca^2+^ signaling likewise plays an important role in regulating autophagy. ROS has been described to induce the transport of the stromal interaction molecule 1 (STIM1) to the plasma membrane, where it activates the store-operated Ca^2+^ release-activated Ca^2+^ (CRAC) channels, resulting in increased Ca^2+^ influx and the activation of calcium/calmodulin-dependent protein kinase kinase 2 (CAMKK2), which in turn activates AMPK and autophagy [71, 72]. Besides, mitochondrial ROS can activate the lysosomal Ca^2+^ channel mucolipin-1 (MCOLN1), resulting in Ca^2+^ release and calcineurin-dependent nuclear translocation of the transcription factor EB (TFEB), which promotes autophagy by inducing Atg and lysosomal gene expression [73] (Figure 3). By contrast, in apparent contradiction, Vlahakis et al. [74] have shown in yeast that during amino acid starvation TORC2-yeast protein kinase 1 (Ypk1) signaling stimulates autophagy by blocking calcineurin activity. Deficiencies in Ypk1 signaling result in mitochondrial respiratory impairment and accumulation of mitochondria-derived ROS that stimulates the Ca^2+^ channel regulatory protein midline 1 (MID1) and activates calcineurin (Figure 3), thereby inhibiting the general amino acid control (GAAC) response and autophagy following amino acid starvation [74].

An exacerbated ROS generation by dysfunctional mitochondria can ultimately shift its role from the bulk autophagy inducer into a self-removal signal for mitochondria through mitophagy [75] (Figure 1). RNS have also been reported to trigger mitophagy. NO has been shown to induce a PINK1-independent PRKN translocation to damaged mitochondria and promote mitophagy associated with mitochondrial fission, via S-nitrosylation of the dynamin-related protein 1 (DRP1) [76, 77]. In this way, ROS/RNS become a fine mechanism of negative feedback regulation by which autophagy eliminates the source of oxidative stress and protects the cell from oxidative damage; hence, it is not surprising to find impaired mitophagy underlying many pathological conditions, including neurodegenerative disease, cancer, and aging [78, 79].

## 4. Mitochondrial Redox Regulation of ATG4

Several core ATG proteins, including ATG3, ATG7, or ATG10, have cysteine residues in their catalytic sites that may be susceptible to oxidative modifications [80]; however, to date, only ATG4 has been reported regulated by ROS. Four ATG4 orthologues (also termed autophagins) have been identified in mammals, namely, ATG4A, ATG4B, ATG4C, and ATG4D, of which the oxidant H_2_O_2_ directly targets and inhibits ATG4A and ATG4B [39].

As stated previously, ATG4 plays a crucial role in the lipid conjugation system of the Atg8 family proteins (Figure 2). The lipidation of MAP1LC3/LC3 homologs is indispensable to normal development of the isolation membrane during the closing step, thereby when overexpression of an ATG4 dominant-negative mutant is used (particularly ATG4B), it results in the accumulation of unclosed isolation membranes [81]. ATG4 is also able to release lipidated MAP1LC3/LC3 from the membrane by catalyzing the deconjugation of MAP1LC3/LC3 [82] (Figure 2). Blocking the deconjugation activity of ATG4 results in defective autophagosome biogenesis [83]. In addition, ATG4 acts maintaining a reservoir of unlipidated MAP1LC3/LC3 by recycling inappropriately lipidated MAP1LC3/LC3 [84]. The coordinated sequence of these processes determines whether autophagy subsequently occurs, and therefore, a precise control of ATG4 activity is needed. Under starvation, a rise of H_2_O_2_ in mitochondria transiently inhibits the proteolytic activity of ATG4, thereby inducing autophagosome formation, presumably by preventing ATG4-mediated deconjugation of MAP1LC3/LC3 during phagophore elongation and closure [39]. A redox control of ATG4 activity has also been reported in yeast [85], in A549 lung carcinoma cells after cadmium exposure [86] and in response to hypoxia and energy stress via the induction of a prooxidant complex composed by the DNA damage-inducible transcript 4 (DDIT4/REDD1) protein, an mTORC1 inhibitor, and the prooxidant thioredoxin-interacting protein (TXNIP) [87]. Suppressed expression of TXNIP, the major endogenous inhibitor of thioredoxins, results in both basal and hypoxia-induced defective autophagy associated with abnormally depolarized mitochondria. Similarly, *redd1* knockout cells show dysregulated ATG4, impaired autophagic flux, and accumulation of defective mitochondria [87]. More recently, studies from our laboratory using APP-PSEN1-SREBF2 mice—a mouse model of Alzheimer's disease that overexpress the sterol regulatory element-binding transcription factor 2 (SREBF2)―have demonstrated that amyloid beta (A*β*) inhibits ATG4 activity and high brain cholesterol levels potentiate this inhibitory effect by reducing the mitochondrial GSH content [88]. The enhanced loss of ATG4 activity shown by cholesterol-enriched cells, which is further potentiated after A*β* exposure, correlates with a greater presence of autophagosomes. However, although autophagy is induced, high cholesterol levels impair autophagosome-lysosome fusion by affecting the proper recycling of key SNARE proteins in the fusion process. Downregulation of the autophagy flux by cholesterol leads to intracellular A*β* accumulation and release, via an unconventional autophagy-mediated secretory pathway [88]. Interestingly, autophagosome synthesis is significantly blunted after treatment with GSH ethyl ester, a cell-permeable form of GSH that recovers the cholesterol-depleted pool of GSH and prevents the oxidative inhibition of ATG4 induced by A*β* [88]. Similarly, resveratrol, a dietary polyphenol with antioxidant and proautophagic properties, has been shown to facilitate the degradation of polyQ huntingtin protein aggregates, the hallmark of Huntington's disease, by regulating ROS-mediated ATG4 activity changes [89]. By recovering ATG4-mediated autophagosome formation, resveratrol protects neuronal-like cells expressing mutant huntingtin from dopamine toxicity [89].

## 5. Mitochondrial Integrity and Dynamics Governs Autophagy

Autophagy requires healthy mitochondria. In yeast, Graef and Nunnari [90] have shown that defects in mitochondrial respiration cause activation of the cAMP-dependent protein kinase A (PKA), a nutrient-sensing regulator that inhibits the induction of *ATG8* expression by amino acid starvation and suppresses the autophagic flux. Later studies further prove the requirement of mitochondrial respiration in the initiation of autophagy in response of energy deprivation, which in turn is regulated by the recruitment and clustering of the mitosis entry checkpoint protein 1 (Mec1, yeast homolog of mammalian ATR serine/threonine kinase) with the energy-sensing sucrose nonfermentating protein 1 (Snf1) and the autophagy-related proteins Atg1 (yeast homolog of mammalian ULK) and Atg13 on the mitochondrial surface [91]. Additionally, a chronic mitochondrial respiration chain deficiency has been reported to affect lysosomal catabolism―with the subsequent accumulation of autophagosomes―by deactivating AMPK and decreasing the activity of the lysosomal Ca^2+^ channel MCOLN1 [92]. Thomas et al. [93] postulate that autophagy can be enhanced by strategies directed to induce a metabolic shift toward oxidative phosphorylation and to increase the mitochondrial metabolism. Their data support a model in which complex I activity, independently of its known contribution to mitochondrial O_2_^•-^ generation, facilitates the transport of phosphatidylserine (substrate for PE biosynthesis) from the ER to mitochondria at mitochondria-associated ER membranes (MAMs), thus favoring autophagosome formation. Autophagy can be induced after mild uncoupling of oxidative phosphorylation by mitochondria-targeted penetrating cations that significantly reduce the mitochondrial *ΔΨ* [94]. Nonetheless, opposite outcomes have also been reported. Overexpression of protein kinase C beta (PRKCB) affects the mitochondrial energy status, lowering the mitochondrial *ΔΨ*, which in turn inhibits autophagy [95]. By contrast, the pharmacological increase of mitochondrial *ΔΨ* counteracts the downregulation induced by PRKCB overexpression and rescues the normal rate of autophagy [95], most likely related to a high proton motive force-induced ROS production [96]. Moreover, perturbations of mitochondrial energy metabolism due to deficiencies of DNA polymerase gamma (Pol*γ*) have been shown to increase O_2_^•-^ and trigger prosurvival autophagy responses via Rictor-mediated mTORC2 activation [97]. In apparent contradiction, cells lacking mitochondrial DNA (mtDNA) have been reported as autophagy-deficient. Lack of mtDNA impairs the signaling pathways mediated by ROS that controls chemical hypoxia-induced autophagy [98]. mtDNA-depleted cells show decreased ROS and impaired ROS-mediated AMPK-ULK1 signaling pathway resulting in reduced autophagosome formation [98, 99]. Also, erythroid cells from aged mtDNA-mutator mice display mitochondrial dysfunction associated with the activation of mTOR and suppression of autophagy, which accelerate the onset of anemia in these mice [100].

Reduced expression levels of the translocase of outer mitochondrial membrane 40 (TOMM40), a key subunit of the translocase of the OMM complex, have been described to stimulate the accumulation of ubiquitin-positive protein aggregates, which are subsequently engulfed by MAP1LC3/LC3-positive membranes [101]. Downregulation of TOMM40 by RNA interference (RNAi) results in reduced proteasome activity and low ATP levels, concomitant with increased ROS levels that lead to the synthesis of unsealed autophagosome-like structures unable to fuse with lysosomes [101]. Noteworthy, these studies show how Tom40 RNAi in *Drosophila* triggers a neurodegenerative process, suggesting a causal link between the maintenance of mitochondrial function, autophagy, and the onset of neurodegeneration.

Regulated changes in mitochondrial dynamics can also determine the cellular response to autophagy. When autophagy is engaged during starvation, mitochondria can elongate due to PKA-mediated inhibitory phosphorylation of DRP1 and the subsequent reduction of fission events [102]. Elongated mitochondria are spared from autophagic degradation and can sustain cellular ATP levels and protect cells from death during starvation. Remarkably, in neurons, recent findings suggest that mitochondrial remodeling associated with early autophagy induction, in addition to prevent cell death, is essential for neuronal differentiation [103].

## 6. Mitochondrial Surface Acts as a Signaling Coordination Hub in Autophagy

Mitochondria are central nodes where autophagic and apoptotic signaling pathways converge [104]. Different proteins present on the mitochondrial surface, including apoptosis-related proteins, are key regulators of autophagy induction that additionally coordinate the cross talk between apoptosis and autophagy. Under normal nutritional conditions, antiapoptotic Bcl-2 suppresses autophagy by interacting with AMBRA1 at the mitochondrial surface and with BECN1 at the ER membranes [105]. Upon starvation, AMBRA1 dissociates from Bcl-2 and binds to BECN1 at ER-mitochondria contact sites to stimulate autophagy [105] (Figure 1). Instead, in response to apoptotic stimuli, BECN1 and other autophagy-related proteins like PI3KC3/VPS34 kinase and ATG4D are cleaved by caspases, upon which they translocate to mitochondria and promote mitochondria-mediated apoptosis [106, 107]. Similarly, calpain-processed ATG5 has been shown to interact with Bcl-xL in mitochondria and induce apoptosis [108]. In cancer cells, the interplay between apoptosis and autophagy induction can also be regulated by the presence of the tumor suppressor p53 and the promyelocytic leukemia (PML) protein in MAMs [109]. The interaction between p53 and PML in these ER-mitochondria appositions regulates the transfer of Ca^2+^ from the ER to the mitochondria, favoring Ca^2+^-dependent apoptosis. In contrast, the absence of p53 or mislocalization of PML out of MAMs activates autophagy in response to cellular stress [109].

MAMs were historically linked to lipid metabolism and Ca^2+^ signaling [110]. Further studies have identified new regulatory roles for ER-mitochondria signaling in different physiological processes ranging from energy metabolism, mitochondrial biogenesis and trafficking, and autophagy [110]. Although the exact mechanisms by which the ER regions come into contact with mitochondria are not completely defined, recent analyses have identified different proteins forming complexes that appear to tether the two organelles. Interestingly, the tightening of ER-mitochondria contacts, via overexpression of VAMP-associated protein B and C (VAPB) or regulator of microtubule dynamics protein 3 (RMDN3/PTPIP51), has been shown to impair rapamycin- and torin 1-induced autophagy [111]. Conversely, the small interfering RNA- (siRNA-) mediated loss of both proteins to loosen ER-mitochondria contacts stimulates autophagosome formation [111]. The mechanism by which VAPB-RMDN3/PTPIP51 complex regulates autophagy likely depends on its role in mediating delivery of Ca^2+^ to mitochondria from ER stores [111]. Disruption of the ER-mitochondria Ca^2+^ communication has also been linked to the activation of the AMPK present at MAMs [112]. AMPK in MAMs can sense the changes of AMP : ATP ratio induced by mitochondrial malfunction, hence becoming activated and phosphorylates BECN1, thus initiating autophagy in a mTORC1-independent fashion [113]. Apparently, at odds with these results, decreased number of autophagosomes has been described after knocking down *Mfn2*, which encodes a mitochondrial outer membrane GTPase that mediates mitochondrial fusion and ER-mitochondria tethering [114]. Furthermore, in cardiomyocytes, MFN2 deficiency has been shown to impair the fusion events between autophagosomes and lysosomes [115]. Completion of autophagy is also reportedly compromised after depletion of sigma nonopioid intracellular receptor 1 (SIG-1R) [116], a MAM-associated chaperone that regulates lipid transport and Ca^2+^ exchange between ER and mitochondria. SIG-1R has been shown to coimmunoprecipitate with key proteins that mediate autophagosome-lysosome fusion, such as ATG14, STX17, and VAMP8. Moreover, impaired autophagosome clearance in SIG-1R knockout cells is recovered after protein reexpression. [116]. Besides these findings, SIG-1R ligands have been found to stimulate ROS production through respiratory complex I [117]. Intriguingly, the same agonists exert a protective effect against mitochondrial oxidative stress induced by the toxic A*β* peptide [117].

## 7. Mitochondria Supply Membranes for Autophagosome Synthesis

Growing evidence suggests that mitochondria are membrane and lipid donor sources for the expansion and maturation of the autophagosomes, most likely through MAMs. Originally, the first organelle proposed as the source of autophagosomal membranes was the ER, through different morphological studies [118, 119]. After that, several membranes have been proposed as sites for the nucleation of the phagophore, including plasma membrane-derived vesicles [120], ER-plasma membrane contact sites [121], Golgi [122], ER-Golgi intermediate compartments (ERGIC) [123], and mitochondria. Based on the transfer of fluorescently labeled lipids from mitochondria to the phagophore, Hailey et al. [114] were the first to postulate the involvement of the OMM in autophagosome biogenesis. Strikingly, their studies showed that the disruption of mitochondria-ER connections dramatically impairs starvation-induced autophagy, by decreasing the lipid transfer from the ER to mitochondria, from where they are ultimately trafficked to the expanding phagophore. Subsequent studies have confirmed the involvement of MAMs in autophagosome assembly [124], by showing that upon starvation STX17 translocates to MAMs where it recruits the preautophagosome proteins ATG14 and ATG5. Then, ATG14 interacts with PI3KR4/VPS15 kinase and BECN1, which are also relocated to MAMs upon starvation, inducing the lipid kinase activity of the PI3KC3 complex, the first step of phagophore formation (Figure 1). Accordingly, knockdown of STX17 blocks the completion of autophagosome formation [124]. The autophagic isolation membrane is unique regarding its high content of unsaturated fatty acids, and deficiencies in stearoyl-CoA desaturase 1 (SCD1), a MAM-enriched enzyme that regulates the ratios of saturated/monounsaturated fatty acids in membranes, have been shown to impair both autophagosome biogenesis and autophagy resolution [125, 126]. More recently, studies by Garofalo et al. [127] have identified the presence of lipid microdomains in MAMs and demonstrate the functional involvement of the lipid raft constituent ganglioside GD3 in the early phases of the autophagic process. The outcomes from these studies show that under starvation-induced autophagy the concentration of GD3 increases within the microdomains, clustering together with the MAM-resident chaperone calnexin, which in turn facilitates its binding with the core-initiator autophagy proteins AMBRA1 and WIPI1. In contrast, knockdown of ST8 alpha-N-acetyl-neuraminide alpha-2,8-sialyltransferase 1 (ST8SIA1), which encodes a synthase involved in ganglioside formation, results in ER-mitochondria contact disruption, with the subsequent hindering of autophagosome nucleation [127, 128].

In addition to sphingolipids, MAMs are enriched in cholesterol. Cholesterol is produced at least in part in MAMs, accounting for the activities of the cholesterol synthetic enzymes being higher in the MAMs than in the ER or mitochondria, respectively [129]. The sterol O-acyltransferase 1 (SOAT1/ACAT1), an enzyme that catalyzes the esterification of membrane-bound cholesterol, is also enriched in MAMs, which leads to the synthesis of cholesterol esters and their subsequent storage in lipid droplets [130]. In steroidogenic cell, cholesterol is transferred from ER to mitochondria for steroid hormone synthesis and the interaction of MAM-resident SIG-1R with the voltage-dependent anion-selective channel protein (VDAC) and the steroidogenic acute regulatory protein (StAR) is reportedly a critical step in this transport [131, 132]. Noteworthy, previous studies from our group in APP-PSEN1 mice have linked increased mitochondrial cholesterol levels to elevated presence of VDAC and SIG-1R in MAMs [133]. Moreover, evidence indicates that changes in the lipid content of MAMs can have a direct impact on ER-mitochondria connection and mitochondrial function, which ultimately may compromise cell viability [133–135]. Indeed, enhanced accumulation of cholesterol within MAMs, associated with an altered MAM architecture and dysfunctional mitochondria, is suggested to contribute to severe pathological conditions such as Alzheimer's disease and the development of obesity-related metabolic syndrome [136–138]. Of note, in many of these pathological settings where perturbations in the ER-mitochondria communication are observed, autophagy is also impaired, thus favoring the hypothesis that disturbances in the MAM action are transitioned into impaired autophagy, given MAM function as operational platforms in the early steps of the autophagosome synthesis.

## 8. Mitochondrial Dysfunction and Autophagy in Human Diseases

Autophagy impairment linked to mitochondrial dysfunction and oxidative stress has been reported in numerous human pathologies. In particular, autophagy involvement in neurodegenerative, cardiovascular, chronic kidney, and liver diseases is well described and extensive reviews can be found elsewhere [139–142]. Although each disease exhibits tissue specific differences, most of them share a loss of autophagy capacities frequently related to aging or chronic exposure to oxidative and inflammatory sources.

Multiple reports indicate that autophagy diminishes with aging and can be a contributory factor to the aging phenotype. Indeed, the expression of ATG proteins and levels of autophagy inducers such as sirtuin 1 are consistently found low in aged tissues [143]. Moreover, mutations of Atg genes prevent the gain of longevity while, conversely, antiaging effects have consistently been observed after stimulation of autophagy by rapamycin or sirtuin 1 [143]. Similarly, the beneficial effect of caloric restriction, the most physiological antiaging intervention that extends life span and delays metabolic and cardiovascular disease onset [144], has been shown to rely in sirtuin 1-dependent activation of autophagy [145]. Mitochondrial dysfunction is also a hallmark of aging [146]. Furthermore, impaired mitochondria have been shown to contribute to age-related pathologies by inducing senescence [147]. Studies from Sun et al. [148], using mt-Keima-expressing mice to measure mitophagy *in vivo*, point to the enfeeblement of mitophagy response to mitochondrial damage as a key contributory factor in mitochondria-driven age-related pathologies. The role of mitophagy in aging and neurodegeneration has further been confirmed by other works [78, 149].

Oxidative stress and mitochondrial dysfunction associated with an accumulation of misfolded protein aggregates are underlying many neurodegenerative disorders such as Alzheimer's disease, Parkinson's disease, amyotrophic lateral sclerosis, Huntington's disease, or frontotemporal dementia [150]. The brain of individuals with these neurodegenerative proteinopathies also displays an accumulation of autophagosome-like structures, suggesting an impaired autophagic flux as the cause of the abnormal disease-specific protein buildups [139]. In line with this notion, *in vivo* studies using mouse models of neurodegeneration showed that genetic inhibition of autophagy enhances degeneration symptoms; conversely, pharmacologic interventions that target autophagic/mitophagic pathways and facilitate the clearance of the neurotoxic aggregates and defective mitochondria display a neuroprotective effect [151].

Similarly to the brain, the myocardium is another highly oxidative tissue where the removal of damaged organelle and particularly of dysfunctional mitochondria through mitophagy is highly relevant. In fact, the progression of cardiovascular pathologies, including atherosclerosis, diabetic cardiomyopathy, or ischaemia-reperfusion-induced damage, has been shown to be affected by autophagy dysregulation [140, 152]. In particular, in diabetic cardiomyopathy, specific autophagic processes seem to operate in the cardiomyocyte, where mitochondria and glycogen particles play an important role [153]. In line with these findings, several reports endorse a detrimental effect of autophagy blockage in diabetes, jeopardizing *β* cells against ER stress in diabetogenic conditions, while suppressing adipocyte differentiation in the adipose tissue [154].

Related to diabetes, but also to other chronic conditions, renal and hepatic pathologies are affected by abnormal authophagy [141, 142]. In the kidney, loss of podocytes, glomerulosclerosis, and damage proximal tubular cells are detected, protecting activated autophagy against apoptosis of tubular cells and enhancing cellular regeneration. Moreover, multiple nephrotoxic medications modify the autophagic efflux [142]. In the liver, the role of autophagy is cell type specific [155]. Nonparenchymal cells such as endothelial cells, resident macrophages (Kupffer cells), or hepatic stellate cells use autophagy for maintaining cellular homeostasis (macrophages, endothelium) or for fueling activation (stellate cells). In hepatocytes, besides homeostatic functions, impaired autophagy is implicated in storage disorders, such as Wilson's disease, metabolic syndrome, or alcohol liver disease [156]. In contrast, in hepatocellular carcinoma (HCC), autophagy contributes to tumor surveillance and, if the tumor arises, in promoting its invasiveness, suggesting a stage-dependent function in liver cancer [155].

Regarding other tumorigenic processes, autophagy has been described to prevent cancer initiation by clearing damaged protein, DNA, and organelles, limiting the oxidative stress and the oncogenic signaling. In contrast, under some circumstances, tumor cells can suffer from nutrient deprivation and hypoxia due to their elevated metabolic demand of growth and proliferation [157]. Therefore, even while for numerous chronic and neurodegenerative disorders autophagy induction seems an interesting approach for treatment, in tumors, autophagy inhibition may be beneficial for cancer therapy, although during a specific therapeutic window [157].

## 9. Concluding Remarks

Autophagy is required for the survival of cells, and the disruption of this process can result in abnormal cell growth or cell death, which may lead to different diseases and pathological conditions. With the onset of aging, autophagy gradually subsides; a similar decline linked to defective mitochondria is also observed in neurodegenerative processes and lysosomal disorders. In contrast, in the context of cancer, although during initial stages autophagy serves as a tumor suppressor, in later stages, the catalytic process protects the tumor cells from the immune system defense mechanisms. Therefore, based on these data, it is clear that a better understanding of the mechanisms that regulate autophagy is needed, which would permit to ameliorate the use of autophagy-modulating therapies that have already been proposed for a variety of disease conditions, as well as sustain longevity. In this review, we have summarized the current knowledge on the role of mitochondria in autophagy, positioning these organelles as central nodes in the signaling pathway for autophagy regulation and highlighting the involvement of mitochondrial oxidative stress. The new insights into the role of the mitochondrial surface as a docking site and a membrane supplier in the first steps of autophagosome assembly have been also outlined. Overall, evidence point out that mitochondria are key players in autophagy regulation, a fact that has to be taken into consideration when handling autophagy for therapeutic use.

## Abbreviations

AMBRA1: Activating molecule in BECN1-regulated autophagy protein 1
AMPK: AMP-activated protein kinase
ATG: Autophagy-related protein
BECN1: Beclin 1
DEPP: Decidual protein induced by progesterone
DRP1: Dynamin-related protein 1
*ΔΨ*: Membrane potential
ER: Endoplasmic reticulum
GABARAP: GABA type A receptor-associated protein
HMGB1: High mobility group box 1
JNK1: c-Jun N-terminal kinase 1
KAT5: Lysine acetyltransferase 5
MAMs: Mitochondria-associated ER membranes
MAPK: Mitogen-activated protein kinase
MAP1LC3: Microtubule-associated proteins 1A/1B light chain 3
MCOLN1: Mucolipin-1
MID1: Midline 1
mTORC1: Mechanistic target of rapamycin complex 1
OMM: Outer membrane of mitochondria
PE: Phosphatidylethanolamine
PINK1: PTEN-induced kinase 1
PI3KC3: Phosphatidylinositol 3-kinase catalytic subunit type 3
PI3KR4: Phosphoinositide-3-kinase regulatory subunit 4
PI3P: Phosphatidylinositol-3-phosphate
PKA: cAMP-dependent protein kinase A
PML: Promyelocytic leukemia
PRKN: Parkin RBR E3 ubiquitin ligase
RB1CC1: RB1-inducible coiled-coil protein 1
RMDN3: Regulator of microtubule dynamics 3
RNS: Reactive nitrogen species
ROS: Reactive oxygen species
SIG-1R: Sigma nonopioid intracellular receptor 1
SNAP29: Synaptosome-associated protein 29
SNARE: Soluble NSF attachment protein receptor
SQSTM1: Sequestosome 1
STX7: Syntaxin 7
STX17: Syntaxin 17
ULK1: unc-51-like kinase 1
UVRAG: UV radiation resistance-associated protein
VTI1B: Vesicle transport through interaction with t-SNAREs 1B
VAMP7: Vesicle-associated membrane protein 7
VAPB: VAMP-associated protein B and C
VDAC: Voltage-dependent anion-selective channel protein
WIPI 1/2: WD repeat domain, phosphoinositide interacting 1/2
Ypk1: Yeast protein kinase 1
ZFYVE1: Zinc finger FYVE-type containing protein 1.
